# Inhibition of HGF/MET signaling decreases overall tumor burden and blocks malignant conversion in *Tpl2*-related skin cancer

**DOI:** 10.1038/s41389-018-0109-8

**Published:** 2019-01-10

**Authors:** Nicole F. Bonan, David Kowalski, Kaitie Kudlac, Kira Flaherty, J. Curtis Gwilliam, Lauren G. Falkenberg, Erik Maradiaga, Kathleen L. DeCicco-Skinner

**Affiliations:** Department of Biology, American University, 4400 Massachusetts Ave, NW, Washington, DC 20016 USA

## Abstract

Tumor progression locus 2 (*Tpl2)* is a member of the mitogen-activated protein kinase kinase kinase (MAP3K) family of serine/threonine kinases. Deletion of the *Tpl2* gene is associated with a significantly higher number of papillomas and cutaneous squamous cell carcinomas (cSCCs). Overexpression of hepatocyte growth factor (HGF) and its receptor MET is abundant in cSCC and can lead to increased proliferation, migration, invasion or resistance to epidermal growth factor receptor (EGFR) tyrosine kinase inhibitors. The aim of this study was to address whether the increased tumor burden in *Tpl2* ^−/^^−^ mice is due to aberrant HGF/MET signaling. C57Bl/6 wild type (WT) and *Tpl2* ^−/^^−^ mice were subjected to a two-stage chemical carcinogenesis protocol for one year. At the time of promotion half of the mice received 44 mg/kg capmatinib (INC 280), a pharmacological inihibitor of MET, in their diet. *Tpl2*^−^^/−^ mice had signficantly higher tumor incidence and overall tumor burden compared to WT mice. Further, carcinogen-intiated *Tpl2* ^−/^^−^ mice could bypass the need for promotion, as 89% of *Tpl2* ^−/^^−^ mice given only DMBA developed papillomas. v-*ras*^Ha^ -transduced keratinocytes and SCCs from *Tpl2* ^−/^^−^ mice revealed an upregulation in HGF and p-MET signaling compared to WT animals. Long-term capmatinib treatment had no adverse effects in mice and capmatinib-fed *Tpl2* ^−/^^−^ mice had a 60% reduction in overall tumor burden. Further, no tumors from *Tpl2* ^−/^^−^ mice fed capmatinib underwent malignant conversion. In summary targeting MET may be a potential new strategy to combat cutaneous squamous cell carcinomas that result from dysregulation in MAPK signaling.

## Introduction

Cutaneous squamous cell carcinoma (cSCC) is the second most common form of cancer in the United States and has the highest mortality of all non-melanoma skin cancers^1^. Approximately 2–5% of cSCC patients develop metastatic disease associated with recurrence and poor long-term prognosis^1^. Skin carcinogenesis is a complex, multistep process and in vivo mouse models remain among the best established tools to study skin cancer development and progression^2^. In the two-stage skin cancer model, initiation often involves application of the carcinogen 7,12-dimethylbenz[a]-anthracene (DMBA), which induces an A to T transversion in codon 61 of the H-Ras gene, followed by repeated applications of the tumor promoter 12-O-tetradecanoylphorbol-13-acetate (TPA)^2^. Tumors begin to develop within a few months, depending on mouse strain, with a small percentage progressing to invasive SCC^2^.

Tumor progression locus 2 (Tpl2), also called MAP3K8, is a member of the mitogen-activated protein kinase (MAPK) family. *Tpl2* has a divergent role in cancer, working as an oncogene or tumor suppressor gene depending on context or tissue in which the signal is aberrantly altered^3^. Increased *Tpl2* activity has been reported in multiple cancer types, including breast, colon and gastric cancer, nasopharyngeal carcinoma, thymoma, lymphoma, and keratoacanthoma^4–9^. However, *Tpl2* can function as a tumor suppressor in cancers such as cSCC, lung cancer, colitis-associated tumorigenesis, and T-cell lymphomas, suggesting the role of *Tpl2* in carcinogenesis remains unclear^10–14^. In a DMBA/TPA carcinogenesis model, we have previously shown that mice devoid of *Tpl2* have a significantly higher incidence of skin tumor development, associated with increased inflammatory signaling and epithelial proliferation^11,14,15^. Additionally, we found stromal fibroblasts contribute to this tumorigenesis.

Hepatocyte growth factor (HGF) is a key factor in the malignant crosstalk between stromal fibroblasts and the primary tumor^16^. HGF released from fibroblasts acts as a ligand for a tyrosine kinase receptor, MET, on neighboring tumor cells. Following ligand binding and autophosphorylation of MET, a variety of signaling pathways become activated including PI3K/AKT, RAS/MAPK, STAT3, WNT, and NFκB^16^. In addition to HGF/MET paracrine signaling, HGF and MET can be co-expressed in many types of cancers, producing an autocrine loop^16^.

Activation of HGF/MET signaling can contribute to proliferation, survival, invasion, migration, drug resistance, and/or angiogenesis in a wide variety of tumors, and correlates with poor clinical outcome^17^. Abundant MET expression or increased HGF transcript and protein levels are found in human skin SCC^18–20^. Aberrant MET activation can occur through several mechanisms including autocrine/paracrine HGF stimulation, mutational activation, gene amplification or upregulation^16,17,21^.

Because HGF and MET are both implicated in cancer progression, many inhibitors have been developed as targeted cancer therapies, including MET inhibitors, anti-MET monoclonal antibodies, and anti-HGF antibodies^22^. Capmatinib (INC280 or INCB28060) is a highly selective, oral small molecule kinase inhibitor of MET^23^. Capmatinib has shown effectiveness in vitro as a single or combined agent in a variety of cancers^24–30^. It is currently in phase I/II clinical trials for papillary renal cancer, hepatocarcinoma (HCC), non-small cell lung cancer (NSCLC), and other advanced solid tumors, and has shown some clinical efficacy^31,32^. However, to our knowledge capmatinib has not been tested for its ability to block skin tumor formation or malignant conversion of chemically-induced papillomas.

In the current study we sought to identify if dysregulation in HGF/MET signaling is responsible for the increase in tumor formation and progression in mice devoid of *Tpl2*. We found an overexpression of HGF/MET signaling in v-ras^Ha^-transduced keratinocytes and SCCs from *Tpl2* ^−/^^−^ mice. Pharmacological inhibition of MET by capmatinib could decrease overall tumor burden in *Tpl2* ^−/^^−^ mice by 60% and prevent all malignant conversion of benign papillomas to invasive SCC. Unexpectedly, DMBA alone was sufficient for tumor formation in *Tpl2* ^−/^^−^ mice, bypassing the need for a promoter. In summary, heightened HGF/MET signaling is critical for tumor formation and progression in Tpl2 ^−/^^−^ mice.

## Results

### Fibroblast signaling contributes to the hyperproliferative phenotype and in vitro conversion frequency of v-ras^Ha^-transduced keratinocytes

**Fig. 1:**
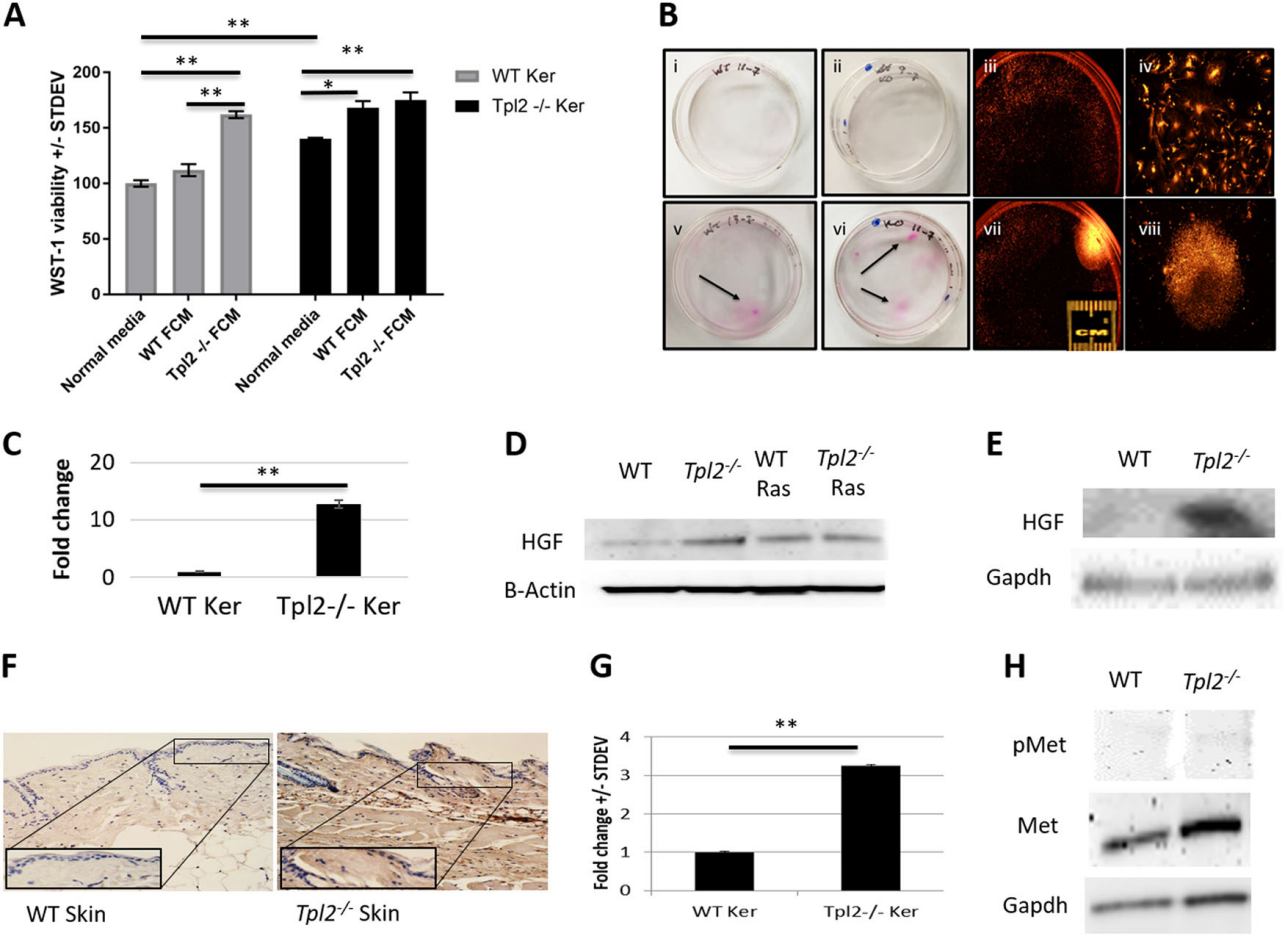
*Tpl2*^−/^^−^ keratinocytes have an increased cell cycle, propensity for malignant conversion, and HGF and MET expression compared to *Tpl2*^+/+^ (WT) cells. **a** WST-1 viability assay for v-*ras*^Ha^-transduced Tpl2^−/−^ and WT keratinocytes (RAS) grown in conditioned media from Tpl2^−/−^ or WT fibroblasts. Results normalized to the v-*ras*^Ha^-transduced *Tpl2*^+/+^ (WT) keratinocyte control. *p < 0.05, **p < 0.01. **b** v-*ras*^Ha^-transduced WT and Tpl2^−/−^ keratinocyte malignant conversion in response to fibroblast signaling. v-ras^Ha^-transduced WT (“i”) or Tpl2^−/−^ (“ii”) keratinocytes cultured in high calcium media develop no foci and remain quiescent in a dispersed monolayer which can be seen at 4X (“iii”) and 40X (“iv”) magnification using rhodamine staining. WT (“v”) and Tpl2^−/−^ (“vi”) keratinocytes cultured in high calcium fibroblast conditioned media form proliferative foci. Foci from rhodamine-stained cultures stain brightly (“vi”, 4X magnification; “vii”, 40× magnification) under fluorescence microscopy. Real-time PCR quantification of HGF in keratinocytes (**c**) and immunoblotting (**d**) of HGF in untreated and v-*ras*^Ha^-transduced Tpl2^−/−^ and WT keratinocytes. Immunoblotting of HGF in fibroblasts (**e**) and immunostaining of HGF in skin (**f**). Real-time PCR quantification (**g**) and immunoblotting (**h**) of p-MET. *p < 0.05

Stromal fibroblasts play a critical role in the growth and progression of SCC. To assess whether *Tpl2* ^−/^^−^ fibroblasts could potentiate growth of *v-ras*^*Ha*^-transduced keratinocytes a proliferation assay was conducted (Fig. 1a). Under normal conditions *v-ras*^*Ha*^-transduced *Tpl2* ^−/^^−^ keratinocytes grew 44% faster than *v-ras*^*Ha*^-transduced WT keratinocytes (Fig. 1a; *p* < 0.01). Additionally, *v-ras*^*Ha*^-transduced WT keratinocytes grown in *Tpl2* ^−/^^−^ fibroblast conditioned media had a 71% increase in cell proliferation compared to *v-ras*^*Ha*^-transduced WT keratinocytes grown in normal supplemented media. *v-ras*^*Ha*^-transduced *Tpl2* ^−/^^−^ keratinocytes grown in *Tpl2* ^−/^^−^ fibroblast conditioned media had a 32% increase in cell proliferation compared to *v-ras*^*Ha*^-transduced *Tpl2* ^−/^^−^ keratinocytes grown in normal supplemented media (Fig. 1a; *p* < 0.01).

To determine whether fibroblast signaling could induce spontaneous, hyperproliferative, neoplastic growth in keratinocytes, an in vitro conversion assay was conducted. Keratinocytes were infected with v-ras^Ha^ retrovirus. Cells that don’t incorporate v-ras^Ha^ retrovirus undergo terminal differentiation in high calcium media. The remaining cells can stay as a monolayer or have the potential to form proliferative foci. *v-ras*^*Ha*^ –transduced WT keratinocytes developed no foci by 8 weeks when grown in normal supplemented high calcium DMEM media (Table 1, Fig. 1b, “i”). However, when WT cells were grown in WT fibroblast conditioned media for 8 weeks 60% of the cultures developed a focus and when WT cells were grown in *Tpl2* ^−/^^−^ fibroblast conditioned media 40% of the cultures developed a focus. However, this fell short of achieving statistical significance (Table 1). *v-ras*^*Ha*^ –transduced *Tpl2* ^−/^^−^ keratinocytes grown in normal supplemented high calcium DMEM media developed no foci by 8 weeks (Table 1, Fig. 1b “ii”). However, 83% of the *Tpl2* ^−/^^−^ cultures developed a single focus in response to signals from WT fibroblasts (Table 1, Fig. 1b, “v”, *p* < 0.005) and 100% of the *Tpl2* ^−/^^−^ keratinocyte cultures grown in conditioned media from *Tpl2* ^−/^^−^ fibroblasts developed at least one focus, with one culture developing two foci (Table 1, Fig. 1b “vi”, *p* < 0.00001). Rhodamine staining was used to differentiate the foci in the cultures from cells in a monolayer.

**Table 1:** *Tpl2* ^−/^^−^ primary keratinocytes infected with v-ras^Ha^ undergo malignant conversion in response to fibroblast signals

Condition	Foci	Ratio of conversion	Significance
WT Ker Ras	0	0/5	
WT Ker Ras in WT FCM	3	3/5	NS
WT Ker Ras in KO FCM	2	2/5	NS
KO Ker Ras	0	0/6	
KO Ker Ras in WT FCM	5	5/6	*P* < 0.005 (vs. KO Ker Ras)
KO Ker Ras in KO FCM	7	7/6	*P* < 0.00001 (vs. KO Ker Ras)

### HGF and MET are upregulated in Tpl2^−/−^ cells

Overexpression of HGF and/or MET can promote tumorigenesis and metastasis. *Tpl2* ^−/^^−^ keratinocytes had twelve-fold higher HGF gene expression (Fig. 1c; *p* < 0.01) and five-fold higher protein levels (*p* < 0.05; Fig. 1d) when compared to WT keratinocytes, although WT and *Tpl2* ^−/^^−^ keratinocytes that had been transduced with *v-ras*^*Ha*^ retrovirus had comparable levels. Fibroblasts from *Tpl2* ^−/^^−^ mice had 8.7-fold higher HGF protein (*p* < 0.05) compared to WT fibroblasts (Fig. 1e). Additionally, HGF was 2.5 fold higher in skin of *Tpl2* ^−/^^−^ mice compared to WT mice (Fig. 1f; *p* < 0.05).

**Fig. 2:**
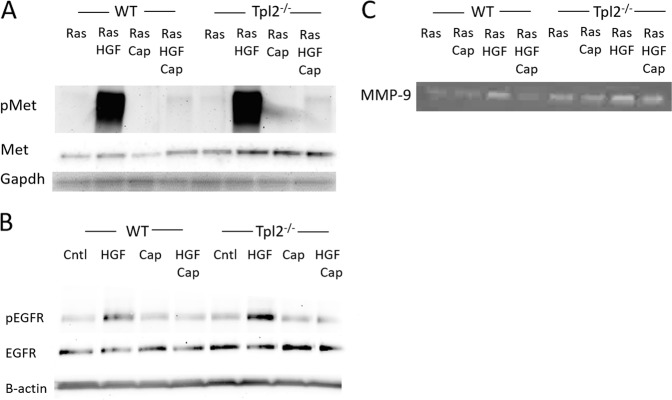
Capmatinib blocks HGF-mediated p-MET expression, reduces MMP-9 activity and prevents phosphorylation of p-EGFR. Primary keratinocytes from newborn mice were cultured in low calcium (0.05 mM) growth media and transduced with *v-ras*^*Ha*^. Cultures were treated with 0.1 nM Capmatinib, followed by administration of 20 ng/ml HGF or vehicle. Total protein was lysed 15 min post HGF treatment. Immunoblotting of p-MET/MET and MET (**a**) p-EGFR/EGFR (**b**) in total cell extracts from primary control keratinocytes was conducted. Lower exposure of (Fig. 2a) is also shown in Supplementary data B. **c** Gelatin zymography of MMP-9 activity in conditioned media from *v-ras*^*Ha*^-transduced Tpl2^+/+^ (WT) and Tpl2^−/−^ (KO) keratinocytes treated with HGF+/− Capmatinib

MET transcripts were 3.1-fold higher in *Tpl2* ^−/^^−^ keratinocytes compared to WT cells (Fig. 1g; *p* < 0.01). p-MET was undetectable in unstimulated WT keratinocytes and barely detectable in Tpl2^-/-^ keratinocytes. In unstimulated conditions total MET protein was 1.8 fold higher in Tpl2^-/-^ keratinocytes (Fig. 1h). In v-ras^Ha^ –transduced keratinocytes HGF stimulation increased p-MET 27 fold in WT keratinocytes and 36 fold in Tpl2^−/−^ cells (Fig. 2a, Supplementary data A, Supplementary data B) compared to unstimulated v-ras^Ha^ –transduced keratinocytes (*p* < 0.01). Moreover, total MET protein was 2-fold higher in Tpl2^−/−^ keratinocytes transduced with v-ras^Ha^ –retrovirus (Fig. 2a).

### Capmatinib blocks MET phosphorylation, MMP-9 activity, and p-EGFR phosphorylation

Capmatinib is a small molecule kinase inhibitor of MET. WT and *Tpl2* ^−/^^−^ keratinocytes were treated with HGF, Capmatinib, or HGF + Capmatinib and protein extracted to confirm the in vitro effectiveness of this drug. Capmatinib inhibited the HGF-stimulated p-MET at concentrations as low as 0.1 nM (Fig. 2a). Recent evidence suggests that MET can interact with EGFR in the skin^20^. Therefore, we tested whether blocking MET phosphorylation with capmatinib can also block p-EGFR. *Tpl2* ^−/^^−^ keratinocytes had two-fold higher p-EGFR compared to WT keratinocytes, both under basal conditions and when stimulated with HGF (Fig. 2b). Treatment with capmatinib fully blocked the HGF-mediated increase in p-EGFR, restoring levels to baseline conditions, yet had no effect on total EGFR protein (Fig. 2b).

Upregulation in MET can increase Matrix Metalloproteinase 9 (MMP-9) production by tumor-associated stromal cells, and blocking MET can significantly reduce MMP-9 activity^33,34^. Thus, we measured MMP-9 activity in *v-ras*^*Ha*^-transduced WT and *Tpl2* ^−/^^−^ keratinocytes. MMP-9 activity was 2.3 fold higher in conditioned media from *v-ras*^*Ha*^-transduced *Tpl2* ^−/^^−^ keratinocytes compared to *v-ras*^*Ha*^-transduced WT cells both under basal conditions and when stimulated with HGF. (Fig. 2c). Capmatinib treatment decreased HGF-induced MMP-9 activity in WT keratinocytes 4-fold and *Tpl2* ^−/^^−^ keratinocytes two-fold.

### Tpl2^−/−^ primary keratinocytes are refractory to TGFβ mediated growth inhibition despite having normal levels of TGFβ receptors and SMAD proteins

**Fig. 3:**
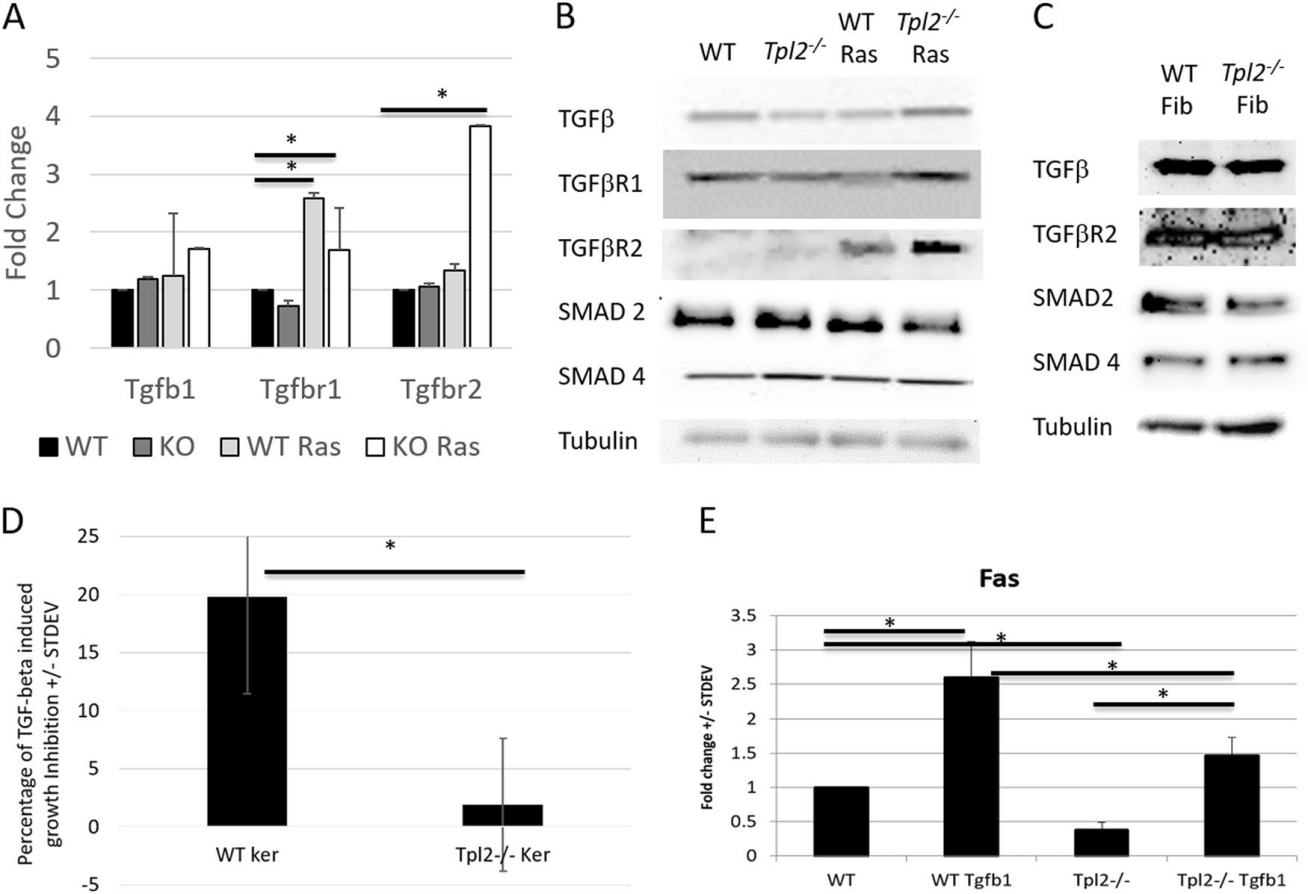
Tpl2^−/−^ primary keratinocytes are refractory to TGFβ mediated growth inhibition despite having normal levels of TGFβ receptors and SMAD proteins. Primary keratinocytes were cultured in low calcium (0.05 mM) growth media or transduced with *v-ras*^*Ha*^ (RAS). Real-time PCR (**a**) and Western analyses (**b**) were conducted for TGFB, TGFBRI and TGFBRII, SMAD2 and SMAD4. **c** Fibroblast protein was collected from newborn WT and Tpl2^−/−^ mice and used for Western analysis. All expression was normalized to a housekeeping gene. **d** WT and Tpl2^−/−^ keratinocytes were treated with 10 ng/ml TGFβ and after 24 h, proliferation was determined using a WST-1 assay. **e** Whole mRNA was isolated and real-time PCR performed and analyzed for relative abundance of Fas mRNA

In some cancers a defect in TGFβ signaling can result in overexpression of HGF^35^. To assess whether the increase in HGF signaling in *Tpl2* ^−/^^−^ keratinocytes correlates with a defect in TGFβ signaling we measured levels of TGFβ1, TGFβR I and II, and associated SMAD proteins (Fig. 3a, b). While TGFβ1 protein levels were 2-fold lower in *Tpl2* ^−/^^−^ keratinocytes (*p* < 0.05), there was no significant defect in TGFβR I and II, SMAD 2 or SMAD 4 proteins in keratinocytes or fibroblasts. (Fig. 3b, C) Interestingly, *v-ras*^*Ha*^-transduced *Tpl2* ^−/^^−^ keratinocytes had an increase in TGFβ, TGFβRI and TGFβRII gene expression (Fig. 3a, *p* < 0.01) and ten-fold higher TGFβRII protein levels compared to untreated keratinocytes.

TGFβ is a potent inhibitor of proliferation in most cell types^36^. Thus, we assessed the ability of TGFβ to inhibit proliferation of WT and *Tpl2* ^−/^^−^ keratinocytes. Within 24 h WT keratinocytes exhibited a 20% decrease in proliferation in response to TGFβ, while *Tpl2* ^−/^^−^ keratinocytes exhibited no growth inhibition (Fig. 3d; *p* < 0.05). The apoptosis-inducing protein Fas is typically overexpressed in TGFβ -stimulated cells, as it participates in TGFβ induced growth arrest. *Tpl2* ^−/^^−^ keratinocytes exhibit decreased transcription of *Fas* relative to wild-type keratinocytes, both when grown under basal conditions and when grown in the presence of TGFβ (Fig. 3e; *p* < 0.05)

### Pharmacological inhibition of MET decreases skin tumor formation in Tpl2^−/−^ mice

**Fig. 4:**
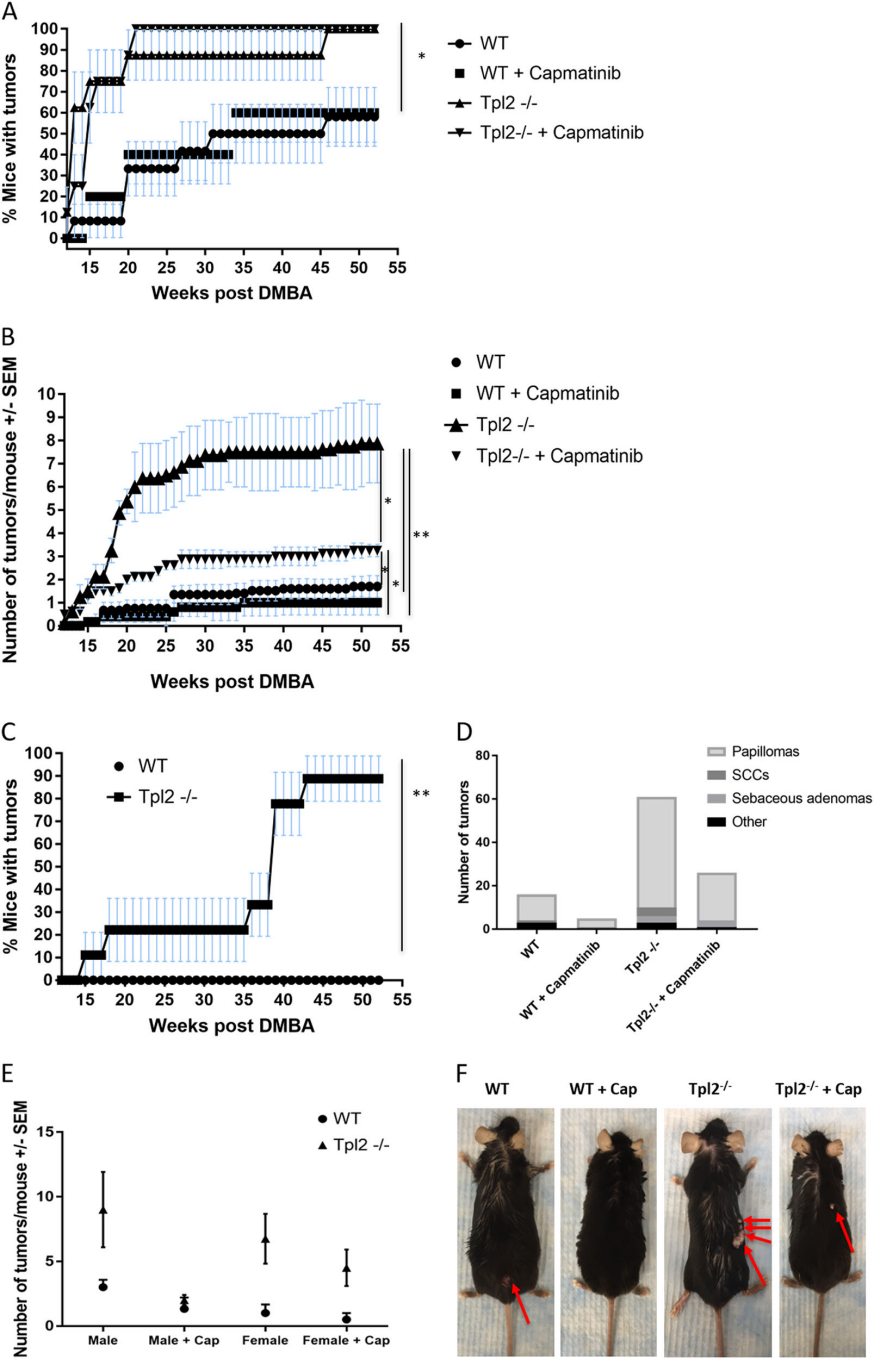
Capmatinib blocks tumor formation and malignant conversion in Tpl2^−/−^ mice subjected to two-stage chemical carcinogenesis. Mice were treated once with 100μg DMBA/200μL acetone. Promotion with 10μg/200μL TPA was started 1 week later and continued for 20 weeks. At the time of promotion half of each genotype received 44 mg/kg capmatinib in their diet. Tumors were measured weekly. The percentage of tumor-bearing mice (**a**) and the number of tumors per mouse (**b**) are plotted vs. time for each group. One-proportion confidence intervals (**a**) and SEM (**b**) were used to generate error bars. Significant differences between curves appeared by week 20 and remained significant until the termination of the study. Significant differences were determined by two-way ANOVA (*p* < 0.001). **c** The percentage of mice receiving DMBA only (no promotion with TPA) are plotted vs. time and significant by week 20 (p < 0.05) and highly significant (*p* < 0.0001) by week 40. Error bars were calculated using one-proportion confidence intervals. **d** Summary of histological examination from the two-stage carcinogenesis experiment. **e** Tumor number and responsivity to capmatinib in males vs. females. **f** Photograph of representative DMBA/TPA mice

To determine if upregulation of HGF/MET signaling contributes to an increased incidence of tumor formation in *Tpl2* ^−/^^−^ mice, a two-stage chemical carcinogenesis model was employed. Hundred percent of *Tpl2* ^−/^^−^ mice subjected to two-stage chemical carcinogenesis developed skin tumors, compared to 58% of WT animals (Fig. 4a). The tumor latency was significantly (*p* < 0.002) different between genotypes by week 13, with 63% of *Tpl2* ^−/^^−^ mice developing papillomas at week 13 in comparison to 8% of WT mice. Further, the number of papillomas was strikingly different between genotypes (Fig. 4b; *p* = 0.001). *Tpl2* ^−/^^−^ mice on normal diet averaged nearly 8 papillomas/mouse and the WT averaged 1.4 tumors/total number of DMBA/TPA WT mice (or 2.28 tumors/mouse if analysis only includes tumor-bearing WT mice). *Tpl2* ^−/^^−^ mice fed a capmatinib diet had a 60% reduction in tumor burden, averaging 3 papillomas/ *Tpl2* ^−/^^−^ mouse (Fig. 4b and Fig. 4f; *p* = 0.02). As expected, no WT control mice receiving only acetone, TPA or DMBA developed tumors. In a similar fashion no acetone-only or TPA-only *Tpl2* ^−/^^−^ mice developed tumors. However, 88.9% of *Tpl2* ^−/^^−^ mice treated only with DMBA developed a papilloma on their skin (Fig. 4c; *p* < 0.0001).

### Capmatinib prevents malignant conversion in Tpl2^−/−^ mice

Tumors underwent a histological examination by a pathologist to determine phenotype and progression. Of the WT mice fed normal diet there were a total of 16 tumors. Twelve of the tumors were papillomas, with one converting to a squamous cell carcinoma (Fig. 4d). Three additional tumors were cutaneous lipomas. This is in comparison to *Tpl2* ^−/^^−^ mice which had a total of 61 tumors, 51 papillomas, four SCCs, three sebaceous adenomas, and three lipomas. In contrast, no *Tpl2* ^−/^^−^ mice fed capmatinib diet had papillomas convert to SCCs (Fig. 4d). Although *Tpl2* ^−/^^−^ mice develop an overall higher tumor burder, there were no statistical differences in tumor size between genotypes and the rate of malignant conversion (7.8 vs. 8.3%) was similar between *Tpl2* ^−/^^−^ and WT mice on normal diet. However, the rate of malignant conversion between *Tpl2* ^−/^^−^ mice on normal diet (8.3%) vs. *Tpl2* ^−/^^−^ mice on Capmatinib diet (0%) was significantly different (*p* < 0.01). In both genotypes male mice developed more tumors than female mice (Fig. 4e; *p* < 0.05).

### SCCs from Tpl2^−/−^ mice have increased expression of HGF and p-MET

**Fig. 5:**
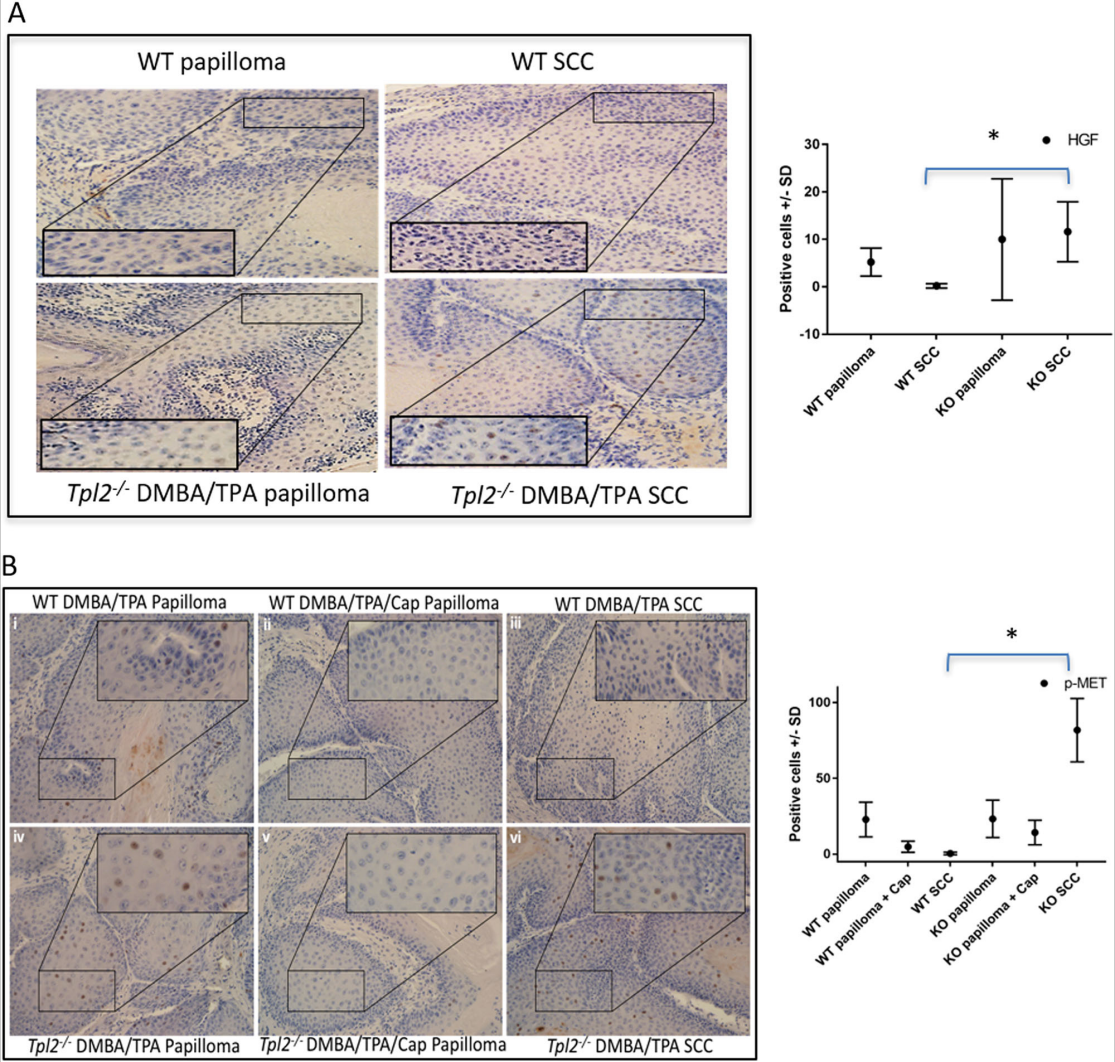
HGF and p-MET are increased in SCCs from Tpl2^−/−^mice. The number of HGF (**a**) and p-MET (**b**) positive cells were counted in five randomly selected regions for each slide and repeated a minimum of three times. Representative photomicrographs at 200× magnification are shown and plotted graphically

We measured HGF and p-MET expression in papillomas and SCCs from WT and *Tpl2* ^−/^^−^ mice. HGF (Fig. 5a) and p-MET (Fig. 5b) levels did not differ statistically between WT and *Tpl2* ^−/^^−^ papillomas. However, both HGF and p-MET expression was significantly (*p* < 0.05) elevated in SCCs of *Tpl2* ^−/^^−^ mice compared to WT SCCs (Fig. 5a, b). Additionally, papillomas from mice fed capmatinib diet had a reduction in p-MET levels (Fig. 5b). Expression of p-MET in SCCs from capmatinib-fed *Tpl2* ^−/^^−^ mice could not be measured, as no *Tpl2* ^−/^^−^ mice fed capmatinib had papillomas convert to SCCs.

## Discussion

The incidence of cutaneous SCC has sharply risen over the last two decades; thus, so has the need to identify driver mutations and aberrantly regulated signaling pathways. MAPK signaling is frequently overexpressed in SCC, therefore the use of kinase inhibitors as potential anti-cancer agents has been attractive. However, inhibition of MAPK signaling has often led to complicated and unexpected findings such as development of de novo mutations and activation of MAPK-dependent and independent bypass pathways, leading to epithelial proliferation, development of squamous cell carcinomas and/or drug resistance^11,37,38^.

We have previously reported that a MAP3K family member, *Tpl2*, can work as a tumor suppressor gene in skin, as *Tpl2* ^−/^^−^ mice develop a significantly higher number of papillomas and cSCCs^11,14,15^. Contributing to this carcinogenesis we have found that stromal fibroblasts have a marked influence on the hyperproliferative phenotype and angiogenic capabilities of *v-ras*^*Ha*^-transduced keratinocytes^11,15^. Fibroblasts secrete a variety of signals, including the growth factor HGF, that are integral to the development and progression of SCC. We measured expression of HGF in keratinocytes, fibroblasts, skin and tumor sections from *Tpl2* ^−/^^−^ mice and found it to be significantly higher than WT controls. This is in agreement with others who reported an increase in HGF production in *Tpl2*-deficient intestinal myofibroblasts^12^.

HGF, when bound to MET, employs several mechanisms to induce cell migration and invasion^17^. One such mechanism is induction of matrix metalloproteinases such as MMP-9^39^. We found considerably higher MMP-9 activity in conditioned media from *Tpl2* ^−/^^−^ keratinocytes, both in untreated *v-ras*^*Ha*^*-*transduced keratinocytes and upon stimulation with HGF. In gastic cancer cells HGF can induce MMP-9 through upregulation in lipocalin-2 and activation of NFκB^39^. Notably both of these factors have been previously shown by our laboratory to be elevated in *Tpl2* ^−/^^−^ keratinocytes^11,40^.

The HGF gene is subject to both positive and negative regulation. Defects in TGFβ signaling can cause an overproduction of HGF, contributing to tumorigenesis^35^. TGFβ is normally secreted by fibroblasts and carcinoma cells and can initiate multiple downstream signaling pathways including those mediated by SMAD proteins. In skin, the role of TGFβ is complicated and paradoxical^36^. Under normal conditions TGFβ is tumor suppressive by serving as a negative regulator of proliferation. Loss of TGFβ and/or its receptors or associated SMAD proteins is associated with tumorigenesis and an increased risk of malignant conversion^41^. However, as cancer advances and cells become refractory to the inhibitory effects of TGFβ, TGFβ can be tumor promoting by stimulating epithelial to mesenchymal transition (EMT), weakening immune surveillance, and enhancing tumor proliferation and chemoresistance^36^. A wide body of literature has implicated RAS signaling in the TGFβ switch from tumor suppressor to tumor promoter, as RAS and TGF beta family members can crosstalk at several different levels^42^.

In this paper we found two-fold lower TGFβ1 protein levels in untreated keratinocytes from *Tpl2* ^−/^^−^ mice but no defect in TGFβ receptors or associated SMAD proteins. However, this defect doesn’t appear to be responsible for the faster cell cycle (Fig. 3d) or the increased HGF (unpublished findings) in *Tpl2* ^−/^^−^ keratinocytes, as exogenous TGFβ1 could not reverse the effect.

In contrast to negative regulation of HGF by TGFβ, multiple hormone responsive and transcription factor binding elements on the promoter of HGF can positively activate transcription of this growth factor^43^. Among the strongest inducers of HGF gene expression in fibroblasts are inflammatory mediators such as prostaglandin E2, synthesized by cyclooxygenase-2, and IL-1β^44,45^. Although we have yet to determine which, if any, factors are responsible for the upregulation of HGF in our system PGE2, COX-2 and IL-1β have all been previously reported by our laboratory to be significantly elevated in *Tpl2* ^−/^^−^ keratinocytes and/or skin, and thus may be good candidates to explain this upregulation^11,15^.

In cancer, HGF binding to MET can be paracrine or autocrine, as HGF produced by stromal fibroblasts can bind to MET on neighboring cancer cells, or cancer cells can produce both HGF and MET. We found elevated p-MET in *v-ras*^*Ha*^*-*transduced keratinocytes, papillomas, and SCCs from *Tpl2* ^−/^^−^ mice. To assess whether pharmacological inhibition of MET could prevent skin tumorigenesis and/or progression in *Tpl2* ^−/^^−^ mice we conducted a DMBA/TPA skin carcinogenesis protocol. Capmatinib is a selective MET inhibitor currently in Phase I/II studies for a variety of cancers^31,32^. However, to the best of our knowledge it hasn’t been studied in cutaneous SCC or a long-term mouse study. We found that capmatinib treatment for 52 weeks in both genotypes was well tolerated, as no adverse effects on body weight or overall health were found (data not shown). Capmatinib treatment reduced overall tumor burden by 60% in *Tpl2* ^−/^^−^ mice. More significant was the finding that all malignant conversion of benign papillomas to invasive SCC in *Tpl2* ^−/^^−^ mice was prevented in mice fed a capmatinib diet, suggesting MET plays an integral role in skin cancer progression. In both genotypes, more males developed tumors than female mice, but *Tpl2* ^−/^^−^ males responded better to capmatinib. However, since sex differences were not the major objective of this work, estrus cycles in female mice were not synchronized and thus could have influenced individual variations between females^46^.

Recent evidence suggests that EGFR is an essential factor for MET-driven skin carcinogenesis^20^. In the presence of a strong promoter, MET activation in the absence of Hras mutations is sufficient to drive skin tumorigenesis^20^. However, MET-driven tumors regress when EGFR is blocked. We add support to the notion that MET and EGFR cooperate in skin carcinogenesis, as capmatinib treatment could block the HGF-stimulated upregulation in p-EGFR.

One of the more interesting results we found in this study was that 89% of *Tpl2* ^−/^^−^ mice treated solely with the carcinogen DMBA developed papillomas, all of which stayed small and didn’t undergo malignant conversion. As expected no WT mice receiving DMBA alone developed papillomas. The Hras gene is a major target of DMBA^2^. Typically, a pro-inflammatory tumor promoter such as TPA is necessary to induce tumorigenesis. Mechanistically, tumor promoters work by inducing a number of inflammatory factors including IL-1 alpha, COX-2, PGE2, and EP2. These factors play a central role in promotion, as DMBA/TPA induced tumor incidence and progression is lower in mice devoid of these genes^47–49^. We have previously reported that skin from *Tpl2* ^−/^^−^ mice have increased epidermal thickness marked by significantly higher levels of neutrophils, macrophages, prostaglandins/prostaglandin receptors, and pro-inflammatory cytokines^11,15^. It is likely that the elevated inflammatory state in *Tpl2* ^−/^^−^ mice can compensate for a promoter, as DMBA only was sufficient for tumor formation. However, the mechanism by which this occurs is still under investigation.

In summary, we have identified the HGF/MET pathway as critical to skin tumor growth and malignant conversion in *Tpl2* ^−/^^−^ mice. This provides new insight into how the MAPK pathway interacts with HGF/MET signaling and highlights the potential of MET inhibitors as therapeutic targets for cutaneous SCC.

## Materials and methods

### Wild type and transgenic mice

Male and female wild type (*Tpl2*^+/+^) and knockout (*Tpl2* ^−/^^−^) C57Bl/6 mice were engineered as previously described^4^. All mice were bred and maintained at The American University animal facility (Washington, DC) under NIH guidelines and an approved IACUC protocol. *Tpl2* ^−/^^−^ status was regularly confirmed by PCR.

### Primary keratinocyte isolation and treatment

Primary keratinocytes and fibroblasts were isolated from *Tpl2* ^−/^^−^ and WT (*Tpl2*^+/+^) mice pups at 1–3 days of age according to standard protocols^50^. Keratinocytes were grown in PromoCell keratinocyte growth media (VWR, Philadelphia, PA) containing hormone supplements, Penicillin-Streptomycin (10,000 U/ml) and low (0.05 mM) calcium. Fibroblasts were maintained in DMEM containing 10% FBS, Penicillin-Streptomycin (10,000 U/ml) and Glutamax (200 mM). For some experiments keratinocytes were infected with v-*ras*^Ha^ retrovirus as described elsewhere^50^. For in vitro capmatinib studies keratinocytes were treated with 0.1 nM capmatinib (INCB28060; Selleck Chemicals, Houston, TX) for 2 h followed by application of 20 ng/mL HGF or vehicle (HGF; R&D Systems, Minneapolis, MN) for 15 min or 24 h prior to protein isolation. For conditioned media experiments, media was collected from treated keratinocytes or fibroblasts 18 h post treatment, centrifuged, and normalized to cell number.

### Quantitative polymerase chain reaction (qPCR)

Total RNA was extracted and purified from cultured primary keratinocytes or fibroblasts using RNeasy Mini kit according to manufacturer’s instructions (Qiagen, Germantown, MD). Complementary DNA (cDNA) was reverse transcribed from template RNA and qPCR was performed using primers obtained from Harvard Primer Bank (Table 2) as previously described^40^. All experiments were repeated a minimum of three times.

**Table 2:** List of primers for real-time PCR

Target	Forward sequence	Reverse sequence	Amplicon length
Tgfb1	CTCCCGTGGCTTCTAGTGC	GCCTTAGTTTGGACAGGATCTG	133
Tgfr1	TCTGCATTGCACTTATGCTGA	AAAGGGCGATCTAGTGATGGA	100
Tgfr2	CCGCTGCATATCGTCCTGTG	AGTGGATGGATGGTCCTATTACA	131
HGF	CCTGACACCACTTGGGAGTA	CTTCTCCTTGGCCTTGAATG	91
MET	CATTTTTACGGACCCAACCA	TGTCCGATACTCGTCACTGC	74
Fas	GCGGGTTCGTGAAACTGATAA	GCAAAATGGGCCTCCTTGATA	61
B-actin	GGCTACAGCTTCACCACCAC	ATGCCACAGGATTCCATACC	214

### Western blotting

Total protein lysates were prepared from keratinocytes and fibroblasts using RIPA buffer containing Halt protease/phosphatase inhibitors in accordance with the manufacturer’s protocol (Thermo Fisher Scientific, Rockford, IL, USA). Twenty-five micrograms of protein was electrophoresed using 4–12% gradient SDS-polyacrylamide gels, transferred to PVDF membrane and blocked with 5% BSA. Primary antibodies for HGF (Abcam; cat #ab83760), TGFβ (Abcam; cat #ab92486), TGFβR1 (Abcam; ab31013), TGFβR2 (Abcam; ab186838), SMAD2 (Abcam; ab63576), SMAD4 (Abcam; ab236321) MET (Cell Signaling; cat #3127), p-MET (Abcam; ab5662), GAPDH (Cell Signaling, Cat #5174), B-actin (Cell Signaling; Cat #4970), tubulin (Cell Signaling, Cat #15115) were incubated with membranes at 1:1000. Anti-rabbit HRP secondary antibodies (Cell Signaling Technology), followed by West Dura Chemilluminescent substrate (Thermo, Rockland, IL) were used for signal detection. Bands were quantified using NIH Image J and normalized to the densitometry for the respective housekeeping gene. All Western blots used pooled protein from triplicate samples and were repeated a minimum of three times. Quantification of Western blots is displayed in Supplementary data A.

### WST-1 cell proliferation assay

5 × 10^4^ v-*ras*^Ha^ –transduced WT or *Tpl2* ^−/^^−^ keratinocytes were plated in quadruplicate in hormone supplemented media or conditioned media from WT or *Tpl2* ^−/^^−^ fibroblasts and incubated at 37 °C in 5% CO_2_. After 24 h, WST-1 was added to each well, incubated, and absorbance read on a microplate reader at 450 nm. Results were normalized to the WT keratinocyte control. For some experiments keratinocyte media was supplemented with 10 ng/mL TGFβ for 24 h.

### Immunohistochemistry

Immunohistochemistry was performed as previously described^15^. Formalin-fixed, paraffin-embedded skin and tumor sections from *Tpl2* ^−/^^−^ and WT mice were processed into paraffin blocks from which 4 μm sections were cut and stained with hematoxylin and eosin (H&E).

Primary antibodies for HGF and p-MET (Abcam; Cambridge, MA, cat # ab83760 and ab5662) and anti-rabbit secondary antibodies (Cell Signaling; Danvers, MA, cat #7074 S) were used. Sections came from a minimum of three individual mice per treatment. As our in vivo experiment only produced 1 SCC in WT mice, we included three additional WT SCC sections from a parallel in vivo study (using the same dosage and timing of DMBA/TPA administration) to increase the number of WT SCCs used for immunohistochemistry. Representative areas were photographed at 200× magnification. For p-MET and HGF staining in papillomas and squamous cell carcinomas the number of positive cells for each section were manually counted in five microscopic fields. For HGF staining in the skin, the optical density (OD) of HGF was quantified using FIJI Image J.

### Zymography

Zymography was performed as previously reported^14^. Briefly, harvested cell-free conditioned media was electrophoresed on 10% tris-glycine gels containing 0.1% gelatin. Zymogram gels were incubated in zymogram renaturing buffer, developing buffer, stained with Coomassie blue G-250, and destained in deionized water. Bands were quantified using NIH Image J.

### In vitro conversion assay

An in vitro conversion assay was modified from Morgan, et al.^51^. Fresh primary keratinocytes were plated in low-calcium (0.05 mM) keratinocyte media and grown to confluence. Cells were v-*ras*^Ha^-transfected for 48 h and transferred to low-calcium keratinocyte media. After 2 weeks cell cultures were maintained in either 0.5 mM high calcium (HiCa) media, HiCa *Tpl2*^+/+^ fibroblast conditioned media, or HiCa *Tpl2* ^−/^^−^ fibroblast conditioned media for 8 weeks while proliferative foci formed. Media was changed twice weekly. At termination, the keratinocyte cultures were stained and fixed with 0.35% rhodamine in formalin, then imaged with a fluorescence microscope.

### Tumor induction experiments

A total of 25 *Tpl2* ^−/^^−^ mice and 29 C57BL/6 WT 8–10 week old mice were initiated with 7,12-dimethylbenz(a)anthracene (DMBA; 100 μg/200 μl acetone) on shaved right dorsal skin. Of these mice, 9/genotype received no further treatment (identified as DMBA only mice). For the remaining sixteen *Tpl2* ^−/^^−^ mice and twenty WT mice, promotion began 1 week later with twice weekly application of TPA painted on the skin (10 μg/200 μl acetone) and continued for 20 additional weeks. Mice were maintained on AIN-93M diet (*Tpl2* ^−/^^−^; *n* = 8 or WT; *n* = 12) or AIN-93M diet containing 44 mg/kg capmatinib (*n* = 8/genotype), a selective c-MET inhibitor. Two additional control groups/genotype received only acetone (*n* = 2) or TPA (*n* = 3). The number and size of tumors were recorded weekly. The numbers of mice required for this experiment are the minimum numbers needed to achieve statistical power. All groups were matched for age, weight and sex. Tumor-bearing animals were individually housed to avoid injury to the tumor sites. Animals were euthanized 52 weeks after the date of initiation, or at an earlier time point if the animal was deemed moribund by the veterinary staff. Portions of skin and tumors were either snap frozen for DNA/RNA/protein isolation or formalin-fixed for IHC. All tumors underwent a histological examination in a blinded fashion by a certified pathologist to determine tumor type (Mass Histology Services, Worcester, MA).

### Statistical analyses

Data was tested for normality, model assumptions were checked, and the data were analyzed with SPSS software. Tumor induction experiments, in vitro conversion assays, zymography, and Western blotting examining genotype and drug effects were analyzed through two-way ANOVA with Tukey’s post hoc test. One-way ANOVA using Fisher’s least significant difference (LSD) post-hoc test was used to analyze qPCR, IHC on skin sections, FAS expression, Western blots that looked at genotype only effects, and viability experiments. Significance for all analyses was assumed at a *p*-value of 0.05 or less. Significance values of *p* ≤ 0.05 are indicated in figures with a single asterisk (*), *p* ≤ 0.01 with a double asterisk (**), and *p* ≤ 0.001 with a triple asterisk (***).

## Supplementary information

Supplementary Data A

Supplementary Data B
