# Supplementary figures and images for: Inhibition of HGF/MET signaling decreases overall tumor burden and blocks malignant conversion in Tpl2-related skin cancer
Source: Oncogenesis. 2019 Jan 10;8(1):1. doi: 10.1038/s41389-018-0109-8 (PMC6328619; doi:10.1038/s41389-018-0109-8)

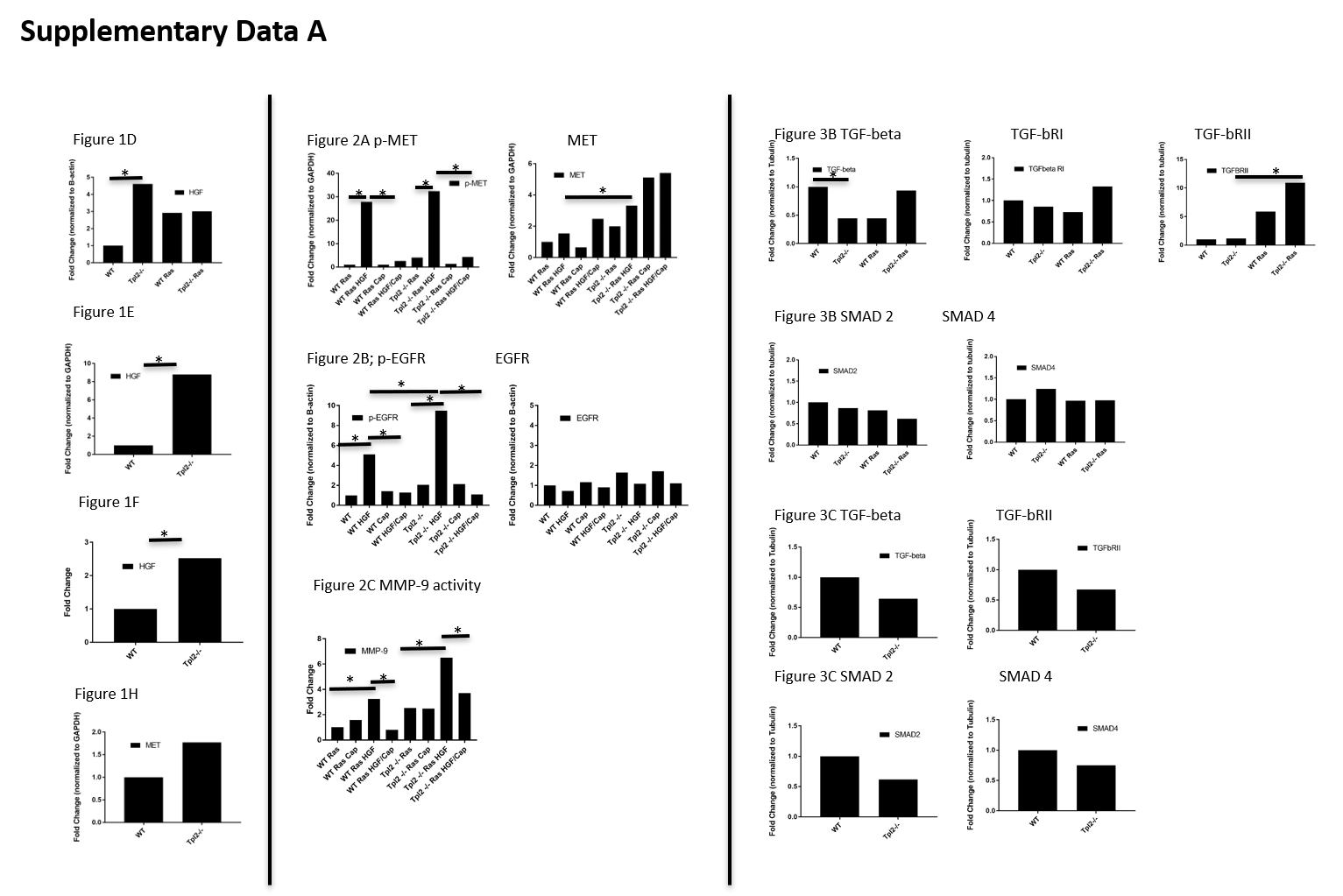

Supplement: Supplementary file 1 — Supplementary Data A [file 41389_2018_109_MOESM1_ESM.tif]

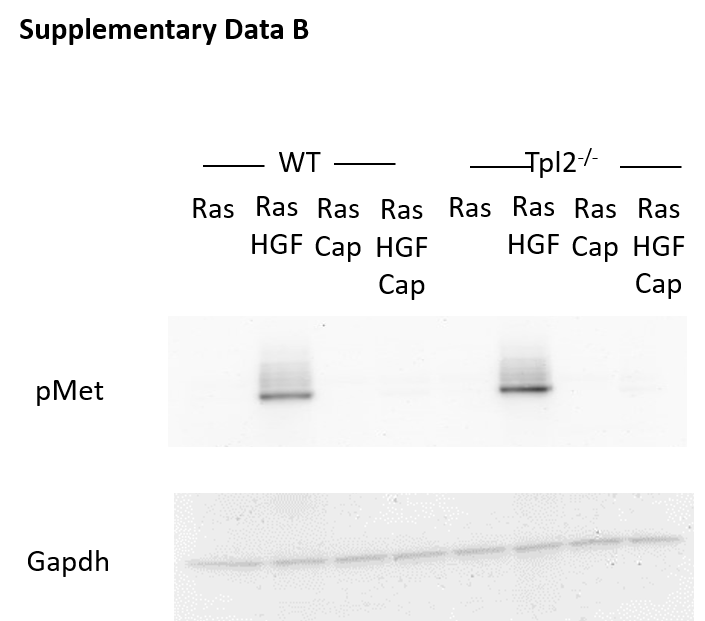

Supplement: Supplementary file 2 — Supplementary Data B [file 41389_2018_109_MOESM2_ESM.tif]
